# Dens Invaginatus—Mandibular Second Molar—Case Report

**DOI:** 10.3390/dj14010027

**Published:** 2026-01-04

**Authors:** Krystyna Pietrzycka, Natalia Lutomska, Cornelis H. Pameijer, Monika Lukomska-Szymanska

**Affiliations:** 1 Department of Endodontics, Medical University of Lodz, 251 Pomorska Str., 92-213 Lodz, Poland; krystyna.pietrzycka@umed.lodz.pl; 2 Platinium Dental Clinic, 2 Gliniana Str., 20-616 Lublin, Poland; 3 Department of Reconstructive Sciences, School of Dental Medicine, University of Connecticut, Farmington, CT 06030, USA; cornelis@pameijer.com; 4 Department of General Dentistry, Medical University of Lodz, 251 Pomorska Str., 92-213 Lodz, Poland

**Keywords:** dens invaginatus, invagination, endodontic treatment, cone-beam computed tomography (CBCT)

## Abstract

**Background:** Dens invaginatus is a rare developmental tooth anomaly that can occur in permanent, primary, and supernumerary teeth, with a tendency to affect the maxillary second incisors. It develops during odontogenesis due to the invagination of the enamel organ into the dental papilla. **Methods:** This study describes the endodontic management of a 24-year-old patient with a type IIIb invaginated tooth in the mandibular second molar. Clinical examination revealed no response to thermal and electrical stimuli, no response to vertical and horizontal percussion, and no pathological mobility. The depth of the gingival pocket was 8 mm. Root canal therapy was performed over three sessions. The patient remained asymptomatic during the treatment and follow-up visits. **Results:** Radiographs at 4-, 6-, and 9-month post-treatment showed healing of the periapical lesion. **Conclusions:** Due to the complex canal anatomy of invaginated teeth, confirming the diagnosis with cone-beam computed tomography (CBCT) is essential. Treating invaginated teeth presents significant challenges for clinicians, requiring a thorough understanding of the dental anatomical variability, advanced manual skills, and the use of specialized equipment.

## 1. Introduction

Anatomical abnormalities in tooth structures are classified as developmental defects caused by issues during the stage of tooth morphological differentiation. An invaginated tooth (dens invaginatus—DI, dens in dente, tooth within a tooth) is one such anomaly [1]. DI was defined by the American Association of Endodontists in the “Glossary of Endodontic Terms” [2] as a developmental defect resulting from the infolding of the crown before calcification. A tooth within a tooth occurs when the enamel-forming organ invaginates into the dental papilla during soft tissue development. During the formation of the hard tissue, a second tooth develops inside the future pulp chamber. It may be clinically visible as an accentuation of the lingual pit in the anterior teeth; in more severe forms, the radiographic appearance resembles a tooth within a tooth, hence the Latin name “dens in dente” [3].

However, the cause of this dental abnormality is not well understood. Several theories have been proposed to explain this phenomenon, including focal growth disorder of the inner enamel epithelium [4], aggressive proliferation of a part of the inner enamel epithelium that invades the dental papilla [5], and the influence of external factors on the tooth bud during its development [6]. Additionally, DI may be linked to cleft lip [7], Wilson’s disease [8,9], chromosomal deletion syndrome (7q32) [10], and other genetic conditions [11,12]. Therefore, the cause of this abnormality remains unknown, and researchers believe that both genetic and environmental factors may play a role [13,14].

The differential diagnosis of a tooth with invagination involves distinguishing it from other conditions with similar clinical or radiographic features [13,15]. These include taurodontism, fusion, C-shaped morphology, supernumerary teeth, and developmental disorders affecting enamel and/or dentin, such as amelogenesis imperfecta, dentinogenesis imperfecta, and dentin dysplasia.

The occurrence of “tooth in a tooth” is a rare morphological abnormality in human dentition. It is said to occur equally in both sexes, which contrasts with other studies where DI was found more often in females [16] or males [17]. A recently published meta-analysis [18,19] indicated a 7.45–9% prevalence of DI on CBCT scans.

In addition, these abnormalities can occur either asymmetrically or symmetrically, alone or with other structural dental disorders, such as microdontia [20], macrodontia [12], hyperdontia [21,22], hypodontia [8,12,23,24], talon cusp [9,25,26], or taurodontism [1]. DI is a rare developmental defect that can affect both the primary [1,8,20,27] and permanent dentition. The most commonly affected teeth are the maxillary permanent anterior teeth, such as the lateral or central incisors [28,29,30,31] and canines [21,31,32,33]. However, the mandibular lateral [9,25,33,34] and central incisors [33,35,36], as well as the mandibular premolars, are also, but less frequently affected. The least affected are the molars [8,16,33,37,38]. Only a few documented cases of permanent molars with DI exist in the literature, including those of the maxillary first molar [39], maxillary second molar [16], maxillary third molar [37], mandibular second molar [40], and mandibular third molar [16]. Consequently, there is a lack of comprehensive clinical studies on the diagnosis and treatment of DI in molars, underscoring the need for further research.

Despite the established Oehlers classification, the current literature on complex DI Type III [21,28,29,30,31,32,41] remains predominantly composed of case reports involving maxillary anterior teeth, likely due to their higher incidence. Consequently, there is a significant gap in knowledge regarding the management of this severe developmental anomaly when it occurs in the posterior dentition—particularly the mandibular second molar, a site that poses notable challenges due to limited access and visibility.

The present case report is notable for providing a comprehensive series that details the management of this rare anomaly, utilizing advanced diagnostic protocols with CBCT as well as contemporary equipment, instruments, and materials. This study offers clinical guidelines for achieving successful outcomes in an anatomically demanding location, thereby expanding the scope of predictable endodontic intervention for Type III DI beyond the traditionally documented anterior cases.

The aim of this study was to present and analyze the endodontic management of a type IIIb invagination in the mandibular second molar.

## 2. Case Presentation

A 24-year-old male with no significant medical history was admitted to the dental clinic with irreversible pulpitis. After administering anesthesia, the pulp cavity was opened, and an antibiotic-steroid paste, Dexadent (CHEMA-ELEKTROMET, Rzeszow, Poland), was placed in the pulp chamber, following the recommendations of the European Society of Endodontology [42,43]. As suggested in the literature, the cavity was filled with a temporary material, glass ionomer cement Fuji IX (GC Europe, Leuven, Belgium) [44]. Due to the atypical anatomy, the patient was referred to the Endodontic Clinic for specialist endodontic treatment.

### 2.1. The First Visit

**Figure 1:**
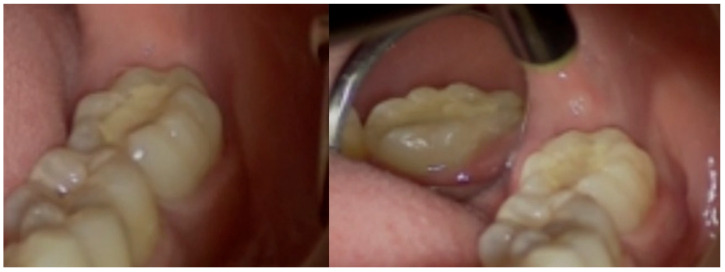
Intraoral photographs of tooth 3.7: (**left**) occlusal and vestibular surfaces; (**right**) occlusal, lingual, and vestibular surfaces (first appointment).

After a week, the patient presented to the Endodontic Clinic. At the last visit, the patient did not report any toothache. Intraoral examination revealed that tooth 3.7 had an unusual anatomical structure, with the crown being elongated unevenly and distally, wide, and featuring seven cusps (Figure 1). In contrast, the anatomy of the crown of tooth 4.7 was normal.

**Figure 2:**
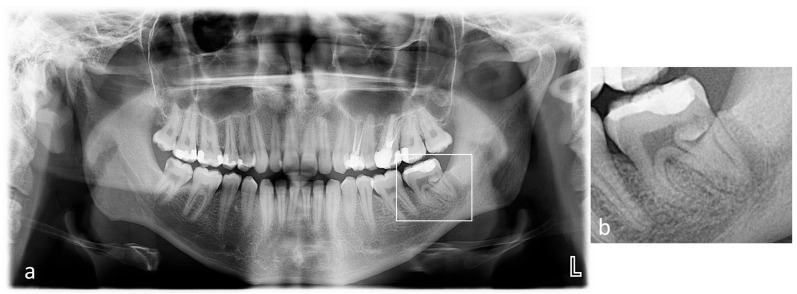
(**a**) Preoperative Panoramic radiograph; (**b**) the magnified panel of tooth 3.7.

No reaction to cold, electrical stimuli, or vertical and horizontal percussion of the tooth was observed during the clinical examination. The mucous membrane around the tooth was normal. However, the depth of the gingival pocket (distally and lingually) was subsequently 8 mm and less than 3 mm, respectively. The remaining pockets measured < 3 mm. No mobility was noted. The patient underwent an OPG radiograph (Figure 2), showing an additional tooth in the distal root of tooth 3.7. Additionally, 3.8 was absent, and the patient reported no extraction in this area. Translucency along the root was distally visible, suggesting thinning of the bone structure. No significant structural changes were observed in the remaining teeth.

Mandibular block anesthesia was administered using 4% articaine hydrochloride with adrenaline 1:100,000 (Citocartin 100, Molteni Stoma, Scandicci, Italy). The temporary dressing was removed under rubber dam. An initial preparation was performed using a long-shank rose-head bur for a slow-speed handpiece, followed by an EndoTracer bur (length 31 mm, size 012) (Komet Dental, Lemgo, Germany). The pulp chamber ceiling was entirely removed using an ultrasonic tip (BUC2 ultrasonic tip; Obtura Spartan, Fenton, MO, USA) under a surgical microscope (magnification 16×). The orifices of the two proximal canals were identified, and DI was detected in the distal canal resting against the distal wall. In front of the DI in the proximal space of the distal canal, there were two canals: one was located lingually, while the remaining endodontic space was blocked by the crown of the DI (diagnosed on CBCT).

**Figure 3:**
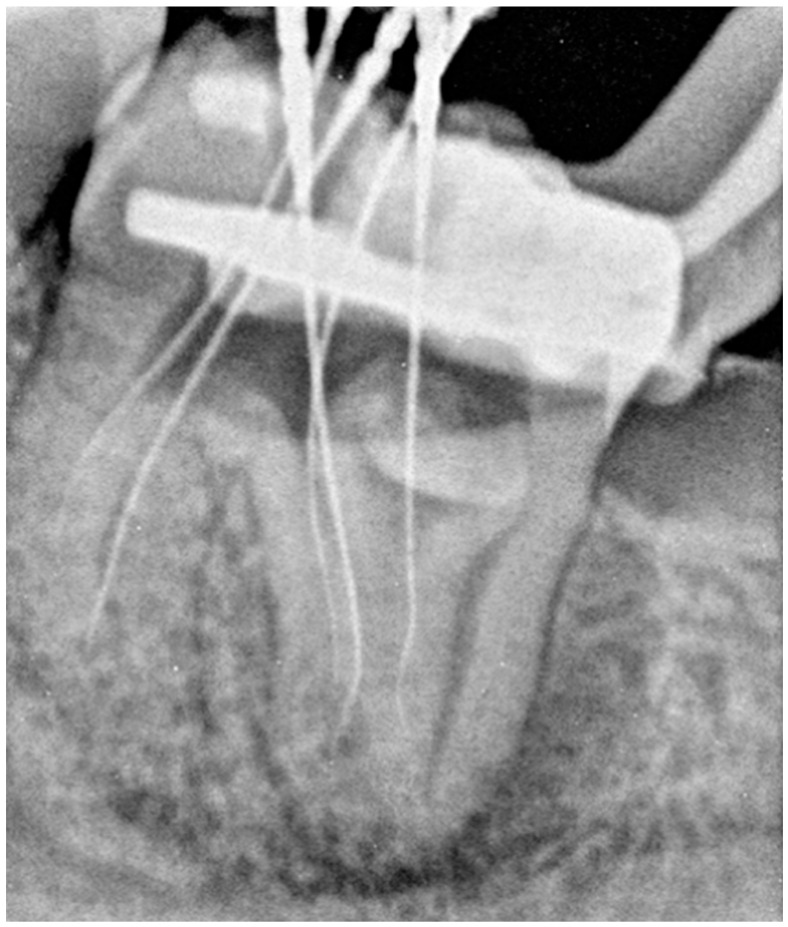
Tooth 3.7—Radiograph with five C-pilots size 10 (the first appointment).

The working length of the canals was measured using an apex locator Raypex 6^®^ (VDW, Munich, Germany) and confirmed radiographically with five file C-pilots of size 10 (VDW GmbH, Munich, Germany) (Figure 3). The canals were then irrigated with 5 mL 5.25% NaOCl solution for each canal. Antibiotic-steroid paste (Dexadent, Chema, Rzeszów, Poland) was applied to the chamber, followed by sterile Teflon, and the cavity was temporarily sealed with Ketac Fil Plus (3M ESPE, Seefeld, Germany). The patient was referred for a CBCT appointment because of the complex anatomy of tooth 3.7.

### 2.2. The Second Appointment

**Figure 4:**
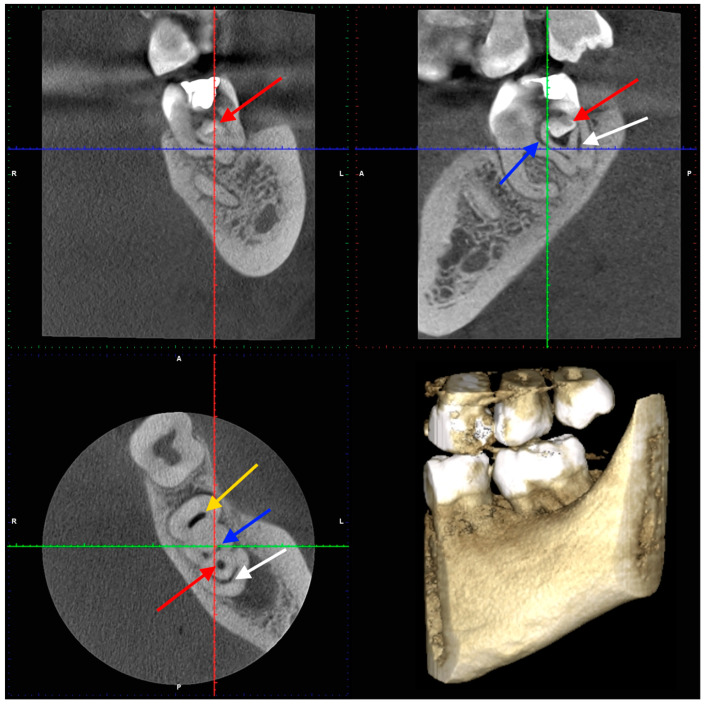
Tooth 3.7—CBCT scans (the second appointment): yellow arrow—mesial canals, blue arrow—two distal canals, white arrow—semicircular space, red arrow—the additional tooth.

**Figure 5:**
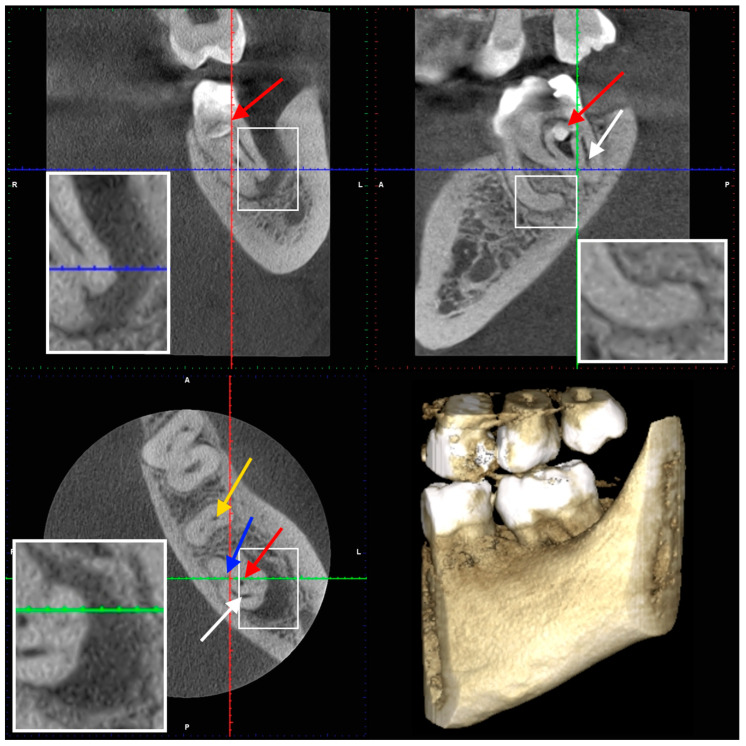
Tooth 3.7—CBCT scans (the second appointment): yellow arrow—mesial canals, blue arrow—two distal canals, white arrow—semicircular space, red arrow—the additional tooth. The magnification of apical regions are visible in the zoomed areas.

**Figure 6:**
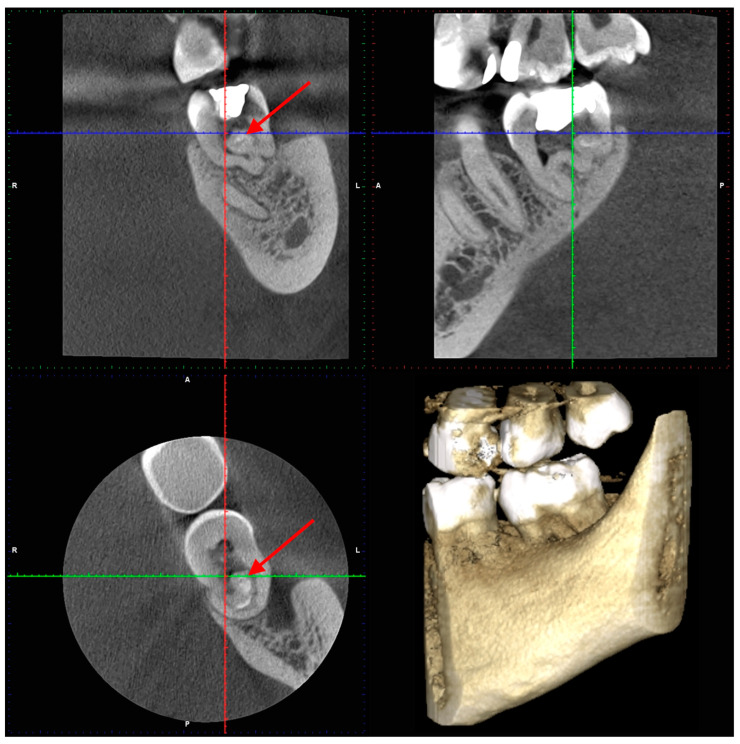
Tooth 3.7—CBCT scans (the second appointment): red arrow—the additional tooth.

After two weeks, the patient returned to the Endodontic Clinic. The patient reported no pain during the last visit. CBCT showed that the mesial root had a severe curvature [45] distally (32.7° and a curvature radius of 3.89 mm) with a Vertucci III root canal configuration [46]. The canals were initially connected, but divided into two separate canals at a depth of 2 mm, only to reconnect after 4.5 mm. The canal lumen disappeared by 2 mm from the apex. In the distal root, there was a tooth within the tooth, with all surrounding tissues (enamel and dentin) and a wide canal. Two distal canals were visible in the horizontal section (in front of the additional tooth) and a semicircular space behind the tooth. Additionally, an extensive inflammatory lesion was observed in the bone at the distal and lingual aspects of the root (Figure 4, Figure 5 and Figure 6).

During the second appointment, under anesthesia and rubber dam isolation, the temporary dressing was removed. Next, the additional tooth in 3.7 was trepanned under microscopic magnification (16×); no vital pulp was present in the chamber. Next, the distal part of the crown was cut using a tapered torpedo thin diamond bur, size 014 (Komet Dental, Lemgo, Germany) and MUNCE Discovery Burs #1 (CJM Engineering Technologies, Santa Clara, CA, USA) to access the blocked space behind the invaginated tooth.

Ultimately, root canal negotiation was performed using C-pilots (sizes: 06–10) (VDW GmbH, Munich, Germany), and the working length of the seven canals was measured using an apex locator RAYPEX 6 (VDW, Munich, Germany). Canal orifices were prepared using the SX ProTaper Gold tool (Dentsply Sirona, Ballaigues, Switzerland). All canals were prepared using the ProTaper Ultimate™ (Dentsply Sirona, Ballaigues, Switzerland) rotary file system (sequence: Slider, Shaper, Finishers F2–F3). The canal in the additional tooth in the distal root was shaped to size F3 (size 30.09), and the remaining canals were shaped to size F2 (size 25.08). The rinsing protocol was as follows: 5.25% NaOCl (CHLORAXiD, Cerkamed, Stalowa Wola, Poland), 5 mL after each file during shaping, 2.5 mL physiological saline, 5 mL 40% citric acid (Cerkamed, Stalowa Wola, Poland) for 1 min, 2.5 mL physiological saline, and 5 mL 5.25% NaOCl for 5 min, followed by 2.5 mL of physiological saline. Disposable plastic syringes (5 mL) with 30-gauge Endo-Eze Tips (Ultradent, South Jordan, UT, USA) were introduced into the canals for irrigation 1 mm short of the working length. An Endo-chuck ultrasonic tip with a K-file size of 20.02 was used to activate the irrigants.

Calcium hydroxide (Calcipast; Cerkamed, Stalowa Wola, Poland) was applied because of a serous exudate in the canals of the distal root. Next, sterile Teflon was placed in the pulp chamber, which was sealed with a temporary filling of Ketac Fil Plus (3M ESPE; Seefeld, Germany). The patient was scheduled for further treatment in two weeks.

### 2.3. The Third Appointment

**Figure 7:**
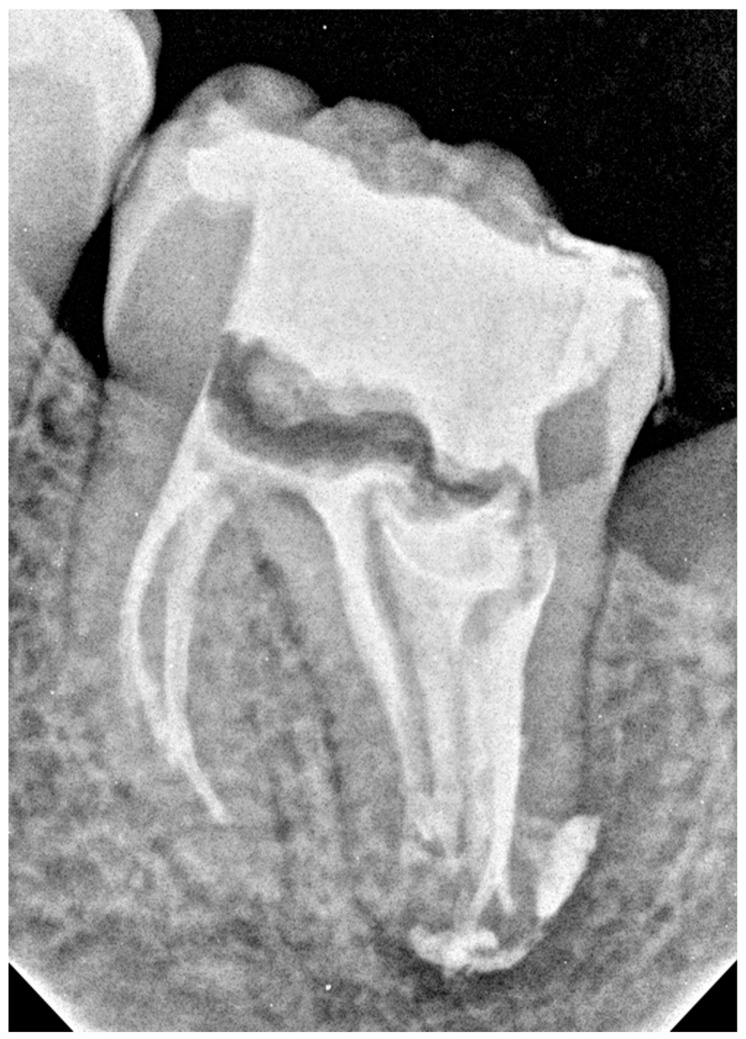
Tooth 3.7: Control RVG image after specialist treatment (the third appointment).

At the third appointment, the tooth was asymptomatic. Under local anesthesia and rubber dam, the temporary dressing was removed, and the rinsing protocol used during earlier visits was repeated with activation using an Endoactivator (Dentsply Sirona, Ballaigues, Switzerland). The canals were dried with sterile paper points (Dentsply Sirona Endodontics, Ballaigues, Switzerland). No exudate was observed in the canals. Gutta-percha points (Dentsply Sirona Endodontics, Ballaigues, Switzerland) recommended for the file system used for preparation were selected. The canals were filled using the continuous wave condensation method with an AH Plus Bioceramic Sealer (Denstply DeTrey GmbH, Konstanz, Germany). The sealer was introduced into the canals using a 24-gauge tip. As described by the manufacturer, the gutta-percha cone sizes F2 and F3 (Dentsply Sirona Endodontics, Ballaigues, Switzerland) were coated with the sealer, inserted into the root canals, and condensed using an up-and-down movement with gentle rotation for better sealer penetration. In wide canals, two or three additional cones were added to improve the sealing. The gutta-percha cones were then cut off using the Gutta-percha cutter of the BeeFill 2 in1 kit (VDW, Munich, Germany). At the orifice level of the two canals in the mesial root and two distal canals in front of the DI, the gutta-percha cones were condensed using size 3/4 cold pluggers (Machtou Pluggers, Dentsply Sirona Endodontics, Ballaigues, Switzerland). In the wide canal of the DI and the semicircular space behind the DI, the cones were cut 3 mm below the orifice and condensed with cold pluggers. The remaining parts of the wide canal and semicircular space were filled with thermoplasticized gutta-percha (150 °C/300 °F) using a pen from the Beefill 2in1 kit (VDW, Munich, Germany) and condensed with appropriate cold pluggers, size 1/2 and 3/4 (Machtou Pluggers, Dentsply Sirona Endodontics, Ballaigues, Switzerland). The pulp chamber was cleaned using alcohol-soaked cotton pellets. Next, the enamel surface was etched with 37.5% orthophosphoric acid (Blue Etch 36%, Cerkamed, Stalowa Wola, Poland) for 15 s, rinsed with water for 5 s, and air dried. G-Premio BOND (GC International AG, Lucerne, Switzerland) was then applied to the enamel and dentin surfaces. After 10 s, the bonding agent was air-dried for 5 s and light-cured for another 10 s. The canal orifices were then filled with white flowable composite resin (ARKONA, Niemce, Poland), and the cavity was temporarily sealed with Ketac Fil Plus glass ionomer cement (3M ESPE, Seefeld, Germany). Finally, a postoperative RVG image showing homogeneously filled canals was obtained (Figure 7). In the mesial root, the material filled the canals up to approximately 2 mm from the radiological apex. In the distal root, the endodontic spaces, including the lateral canals and sealant beyond the apex, appeared filled. The patient was informed of the need for a follow-up appointment, and a final restoration with composite resin was recommended.

### 2.4. The Fourth Appointment

**Figure 8:**
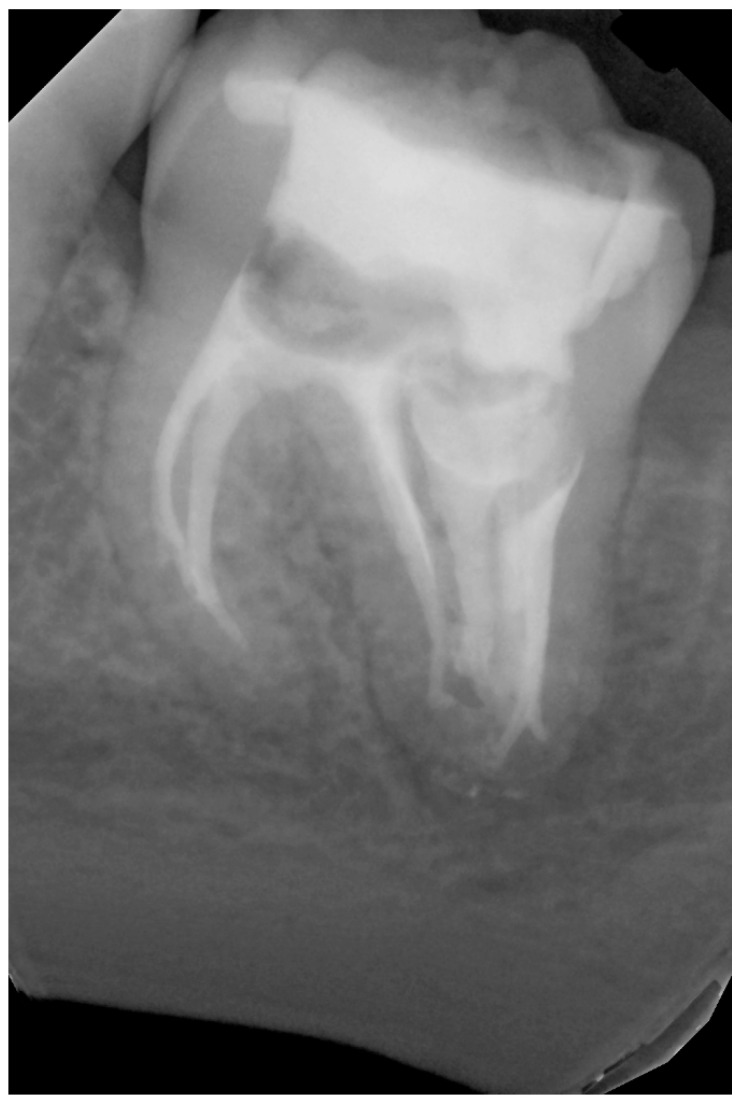
Tooth 3.7, post-operative RVG image taken 4 months after root canal filling (the fourth appointment).

After four months, the tooth remained asymptomatic. The patient visited the hospital because of caries in another tooth. He had not returned for a final restoration, but the cavity appeared tightly sealed with temporary filling material. During the visit, another post-operative radiograph was obtained (Figure 8), showing healing of the lesion adjacent to the distal root. Intraoral examination revealed no changes in the mucous membrane or presence of periodontal pockets (<3 mm). Additionally, normal physiological mobility and a lack of pain on vertical and horizontal percussions were noted. The temporary filling was then removed under rubber dam isolation. After selective enamel etching for 5 s, rinsing, and drying, G-Premio BOND (GC International AG, Lucerne, Switzerland) was applied, allowing it to react for 10 s. Next, air drying for 5 s and light curing for 10 s were performed. The cavity was filled with a 3 mm layer of SDR Plus Refill A2 and a thin layer (1–2 mm) of composite resin Neo Spectra ST HV A2 (Dentsply Sirona, Ballaigues, Switzerland), which were light-cured for 20 s. The patient was scheduled for a follow-up visit after six months.

### 2.5. The Fifth Appointment

**Figure 9:**
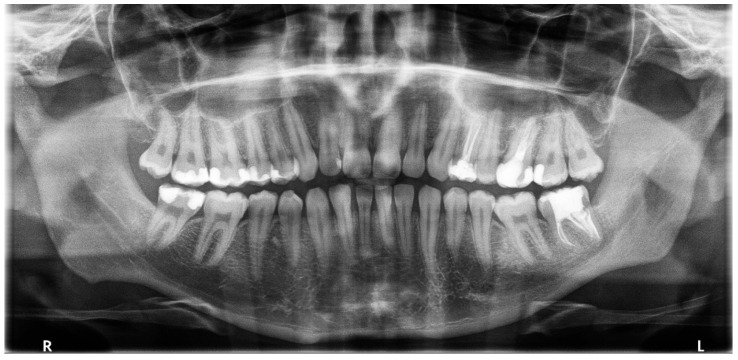
Six-month post-operative OPG radiograph after root canal treatment (fifth appointment).

On the six-month post-operative OPG radiograph (Figure 9), healing of the lesion was observed. The patient reported no pain or provoked pain.

### 2.6. Sixth Appointment

**Figure 10:**
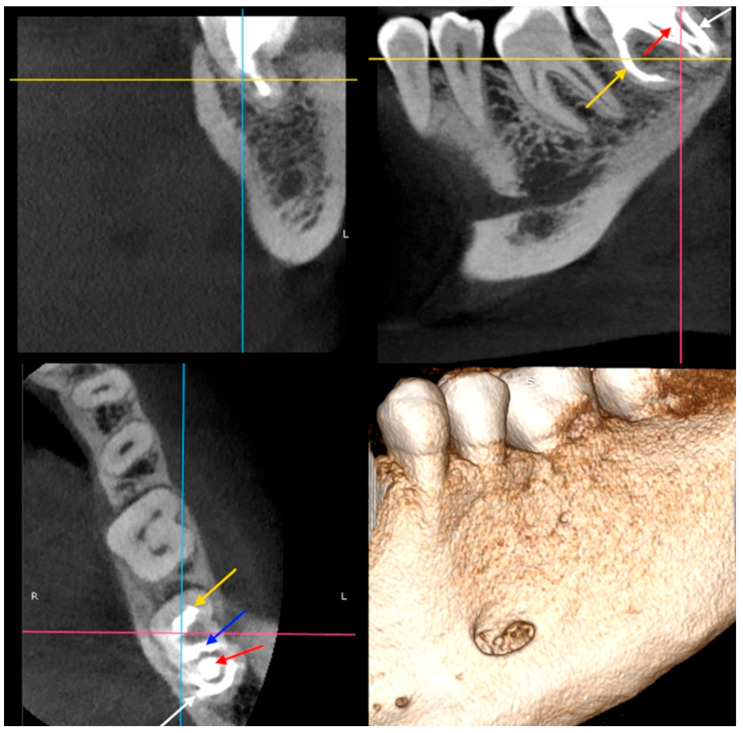
Tooth 3.7—CBCT scans 9 months post-operative: yellow arrow—mesial canals, blue arrow—two distal canals, white arrow—semicircular space, red arrow—the additional tooth.

At the nine-month follow-up, CBCT imaging (Figure 10).demonstrated widening of the periodontal ligament space at the mesial root; additionally, the radiolucent area at the distal root appears to have decreased in size compared with the earlier CBCT images shown in Figure 5 (the second appointment). The clinical evaluation indicated that tooth 3.7 was asymptomatic.

## 3. Discussion

This paper presents a case of radicular invagination in a mandibular second molar, detailing its diagnosis, treatment, and follow-up care. Preoperative CBCT facilitated proper diagnosis, treatment planning, and decisions regarding the treatment procedure [13,47,48]. Similarly, in this and other studies, CBCT was introduced as an additional diagnostic tool [13,49,50,51]. According to the European Society of Endodontology (ESE) and the American Association of Endodontists (AAE), one recommended application of CBCT in endodontics is to confirm the diagnosis of DI and develop a treatment plan [52]. In this case, after a thorough analysis of the CBCT scans, the complex anatomical variation in the invaginated tooth was visualized, which assisted in establishing the treatment strategy.

Different classification systems have been published previously. The most well-known and widely used classification method was proposed by Oehlers [5]. According to the author, three types of invaginations are distinguished based on the extent of the anomaly: Type I, minimal invagination involving only enamel and dentin beginning in the crown and not extending beyond the enamel-cement junction. Type II invaginations penetrate deeper, forming a blind pocket that may or may not communicate with the pulp chamber but remains within the root canal without connecting to the periodontal ligament. The most advanced anomaly is seen in the crown of teeth with Type III invagination. It extends through the root, usually without immediate communication with the pulp, and connects to the periodontal ligament via a second foramen on the lateral surface of the tooth (Type IIIa) or through the apical foramen (Type IIIb). Additionally, a new Type IV has recently been proposed, in which the invagination has lateral communication with the periodontium, and a lateral canal arises from the main root canal [51]. The case presented in this study can be classified as Type IIIb with an enlarged crown, which is typical for teeth with this abnormality [13].

In the literature, cases of type III invagination have been reported in incisors [3,25,29,30,31,35,39,53,54,55], canines [31,32], premolars [31,56], and molars [40]. The structure of DI type IIIb in mandibular molars is rare [16,17]; therefore, this case report should be of particular interest to clinicians and researchers.

Teeth with invagination may not exhibit significant deviations from the normal anatomy, displaying minor deformities, such as an enlarged palatal cusp in the incisors or a complete change in crown shape. Owing to their extensive morphological diversity, these cases are more prone to caries, which can eventually lead to pulpitis and periapical lesions, as seen in the present case. Therefore, preventive management is critical when abnormalities are diagnosed. In all types of invaginations, when a minor invagination involves the crown as a deep pit or foramen cecum, the primary goal is to prevent caries by sealing the invagination with a restoration to prevent bacteria from penetrating the tissues. If hard tissue is destroyed without pulp involvement, removal of caries and composite restoration are necessary [3,13,34]. The treatment approach depends largely on the type of invagination and the extent of hard tissue destruction. If the pulp is infected or periapical tissue is involved, endodontic [3,9,13,30,31,32,57], microsurgical, or surgical treatment [3,8,13,22,30,35,53] should be performed. Teeth exhibiting type I invagination typically do not show significant deformation, allowing root canal treatment (RCT) to be completed in a single visit. Disinfection of the true root canal with intracanal medication is necessary in cases of pulp necrosis and apical periodontitis. For cases involving type II invagination with pulpal involvement and periapical lesions, it is advisable to treat the invagination and main root canal separately, if feasible. In certain clinical scenarios, due to intricate and complex anatomy, uniting the invagination and the main canal may be necessary to gain better access to the apical root canal. Additional strategies should include CBCT examination for diagnosis and treatment planning, magnification with an operating microscope for improved visualization, supplementary disinfection methods, such as ultrasonic tips and intracanal medication, and thermoplasticized obturation. In type III invagination, a periapical lesion may develop alongside a healthy, non-inflamed pulp when infection in the periapical tissue originates from bacterial invasion of the invagination. In such cases, the pulp within the main canal can be preserved, and cleaning, shaping, disinfecting, and filling of the invagination can promote periradicular healing. If inflammation or necrosis occurs in both the main canal and invagination, treating both is essential, utilizing all the previously mentioned strategies [15,31,33].

Endodontic treatment of teeth with diverse morphologies presents a significant challenge and requires an individualized approach for each case [15,40]. The literature describes various treatment options for cases of DI: RCT on the main root and intentional replantation [30,53], apical root resection [35], extraction [22,58], apexification, and RCT with MTA, as well as a recently published novel approach using CBCT and 3D printing technology [59]. In the literature [30,31,60,61,62,63], follow-up periods ranged from 6 months to 50 years, with endodontic treatment success rates for cases diagnosed with DI generally reported as high (80–97%). In instances where primary root canal therapy fails, endodontic retreatment [30,31], either alone or combined with surgical intervention [30,31,61], has been shown to be an effective management approach. During endodontic procedures, magnification [39], ultrasonic tips for preparation, and irrigant activation were used, as in other studies [3,49,64,65], to ensure thorough cleaning and disinfection of the endodontic system with invagination. The final root canal filling was performed using the continuous wave condensation method, and a calcium silicate sealer was used for obturation, as reported in other studies [40,66,67]. During follow-up examinations at 4, 6, and 9 months after RCT, healing of the periapical lesions was observed.

### 3.1. Study Limitations

In this case, the etiology and genetic background are unknown, leaving potential hereditary or developmental factors unexplored. As the study involves a single patient and one tooth, the anatomical, etiological, and therapeutic observations may not be generalizable to other patients, tooth types, or ethnic groups. Therefore, the findings of this single-case study cannot be extrapolated to the broader population of patients with dens invaginatus (DI). Investigations involving larger populations are needed to expand knowledge on this condition.

The follow-up period of nine months may be insufficient to evaluate long-term success or late complications, such as root fracture or recurrent infection. Long-term clinical and radiographic follow-up, ideally for 5–10 years, is recommended to assess the longevity of the treatment and detect delayed complications.

The diagnosis and treatment were performed by an experienced endodontic specialist; thus, the outcomes may not be reproducible by general practitioners. Additionally, the treatment followed a single protocol, limiting comparisons with alternative modalities and reducing the direct applicability of the results to routine practice.

### 3.2. Future Directions

Prospective studies or case series with larger sample sizes are needed, including patients with different types of DI (Oehlers Type I–III) and a variety of treatment approaches (e.g., preventive sealing, MTA apexification, revascularization, surgical intervention, extraction with implant placement). This would help establish evidence-based treatment protocols.

Further research should explore the genetic and molecular basis of DI, ideally in collaboration with geneticists and through the study of families with multiple affected members. Advanced imaging techniques, such as micro-CT, are recommended for future cases to better characterize complex internal anatomy before and after treatment.

Regenerative endodontic procedures and bioactive materials (e.g., Biodentine, iRoot BP Plus) should be incorporated into treatment protocols for teeth with DI and open apices. Finally, standardized classification systems and treatment decision algorithms—integrating 3D imaging findings, pulp vitality status, and root development stage—should be proposed by relevant professional organizations.

## 4. Conclusions

Endodontic management of invaginated teeth requires a personalized approach, as no standardized protocol currently exists. In such cases, referral to a specialist with the appropriate expertise, clinical skills, and access to advanced diagnostic technologies is crucial for effective treatment planning and execution. The use of modern materials, three-dimensional imaging, and surgical microscopy can greatly enhance treatment predictability and contribute to successful outcomes. Establishing standardized endodontic guidelines for managing complex cases like these would be highly valuable.

## Data Availability

The original contributions presented in this study are included in the article. Further inquiries can be directed to the corresponding author.

## Abbreviations

The following abbreviations are used in this manuscript:

CBCT	Cone-Beam Computed Tomography
DI	Dens invaginatus
OPG	Orthopantomogram
RCT	Root Canal Treatment
